# The effect of CGRP and SP and the cell signaling dialogue between sensory neurons and endothelial cells

**DOI:** 10.1186/s40659-024-00538-6

**Published:** 2024-09-11

**Authors:** Alice Leroux, Micaela Roque, Elina Casas, Jacques Leng, Christelle Guibert, Beatrice L’Azou, Hugo Oliveira, Joëlle Amédée, Bruno Paiva dos Santos

**Affiliations:** 1 Univ. Bordeaux, INSERM, BIOTIS, Bordeaux, U1026, F-33000 France; 2 Univ. Bordeaux, CNRS, UMR 5258, Solvay, Pessac, LOF F-33006 France; 3 INSERM, Centre de Recherche Cardio-Thoracique de Bordeaux, U1045, Pessac, F-33604 France; 4 Univ. Bordeaux, Centre de Recherche Cardio-Thoracique de Bordeaux, U1045, Bordeaux, F-33000 France; 5 Univ. Paris Cité, URP2496-BRIO Pathologies Imagerie et Biothérapies Orofaciales, Montrouge, F-92120 France

**Keywords:** Angiogenesis, Calcium signaling, CGRP, Substance P, Innervation, Nitric oxide

## Abstract

Increasing evidences demonstrate the role of sensory innervation in bone metabolism, remodeling and repair, however neurovascular coupling in bone is rarely studied. Using microfluidic devices as an indirect co-culture model to mimic in vitro the physiological scenario of innervation, our group demonstrated that sensory neurons (SNs) were able to regulate the extracellular matrix remodeling by endothelial cells (ECs), in particular through sensory neuropeptides, i.e. calcitonin gene-related peptide (CGRP) and substance P (SP). Nonetheless, still little is known about the cell signaling pathways and mechanism of action in neurovascular coupling. Here, in order to characterize the communication between SNs and ECs at molecular level, we evaluated the effect of SNs and the neuropeptides CGRP and SP on ECs. We focused on different pathways known to play a role on endothelial functions: calcium signaling, p38 and Erk1/2; the control of signal propagation through Cx43; and endothelial functions through the production of nitric oxide (NO). The effect of SNs was evaluated on ECs Ca^2+^ influx, the expression of Cx43, endothelial nitric oxide synthase (eNOS) and nitric oxide (NO) production, p38, ERK1/2 as well as their phosphorylated forms. In addition, the role of CGRP and SP were either analyzed using respective antagonists in the co-culture model, or by adding directly on the ECs monocultures. We show that capsaicin-stimulated SNs induce increased Ca^2+^ influx in ECs. SNs stimulate the increase of NO production in ECs, probably involving a decrease in the inhibitory eNOS T495 phosphorylation site. The neuropeptide CGRP, produced by SNs, seems to be one of the mediators of this effect in ECs since NO production is decreased in the presence of CGRP antagonist in the co-culture of ECs and SNs, and increased when ECs are stimulated with synthetic CGRP. Taken together, our results suggest that SNs play an important role in the control of the endothelial cell functions through CGRP production and NO signaling pathway.

**Supplementary Information:**

The online version contains supplementary material available at 10.1186/s40659-024-00538-6.

## Introduction

Human bone is a fully vascularized and innervated tissue, with a coupling between blood vessels and nerve fibers [1]. Nerve fibers can be found throughout the bone tissue but are more abundant in the periosteum and in highly vascularized and metabolically active regions [2]. Vascularization and innervation are anatomically and functionally coupled in bone tissue, nerve fibers are present along the blood vessels and, at the same time, nerve fibers contain blood capillary networks. Following these observations, we and others have shown that sensory nerves can coordinate some aspects of vascularization and vascular cells [3–6], sharing common mechanisms during their development as well as during the bone regeneration process [4] through a variety of neurogenic or non-neurogenic factors [6].

Indeed, both vascular and nervous tissues share common signaling molecules. Endothelial cells and nerve cells can both secrete angiogenic and neurogenic factors during vessel and nerve formation as well as to maintain their respective functions. In 2017, Yuan et al., reported that human microvascular endothelial cells promote the development of dorsal root ganglion neurons at least *via* BDNF pathway in a co-culture system and the activation of ERK pathway [7]. In turn, sensory nerves can secrete calcitonin gene-related peptide (CGRP) and substance P (SP), both classic sensory neuropeptides, that stimulate endothelial cell proliferation and in vivo angiogenesis [8], and control endothelial cell function. SP also can stimulate nitric oxide (NO) production in endothelial cells and then protect the bone vasculature [9]. However, while multiple factors have been identified in the coupling between innervation and vascularization, there is still a variety of mechanisms of action and signaling pathways in the SNs and ECs interplay that remains yet to be elucidated [10].

In our previous work, we have used microfluidic chambers to mimic the innervation process, where cell interactions were achieved through axons emitted by neurons toward the ECs. These devices also permitted to analyze the two cell populations at proteomic and gene levels and thereafter enabled the evaluation of the influence of sensory neurons (SNs) on the target cells [3, 11]. We have first demonstrated in vitro that rat primary SNs were able to enhance the expression of angiogenic markers in rat bone marrow-derived endothelial cells, and more specifically to stimulate extracellular matrix remodeling [3] through MMPs activities.

Here, we propose to explore the interplay and cell signaling mechanisms on the effect of sensory neurons on endothelial cells. Numerous mechanisms, known as neurovascular coupling, have been explored and were a point of focus in this study. Among them, endothelial Ca^2+^ signaling, constitutes an emerging pathway in the regulation of neurovascular coupling [12]. In this respect, intercellular calcium (Ca^2+^) waves, that represent the propagation of increases in intracellular Ca^2+^ through a cell network, appear to be a fundamental mechanism for coordinating multicellular responses [12]. The calcium propagation predominately involves cell to cell communication, with internal messengers moving *via* gap junctions, including the connexin family, e.g. connexin 43 (Cx43), or other messengers mediating paracrine signaling [13] with a special emphasis on the possible participation of NO and endothelial NO synthase (eNOS) in this process [14].

A second focus has been drawn on the classical sensory neuropeptides released in close proximity to the vascularized tissue, and which can then directly modulate functions of endothelial cells. Previously, we showed that SNs, indirectly co-cultured with ECs, secreted CGRP and SP that could regulate ECs functions [3]. Similar to CGRP, it has long been described that SP induces endothelium-dependent vasodilation through the activation of nitric oxide synthase and thus release of nitric oxide [15–17]. It is thought that angiogenesis in bone is in part the result of SP release and endothelial progenitor cells migration from the bone marrow into the injured peripheral tissue [18, 19]. In addition, it has been shown in different vascular systems that SP-induced vasodilation is mediated by cyclic-GMP signaling through different pathways involving endothelial NO or direct endothelial-independent pathways [20].

In such context, the aim of this study is to further explore the cell-cell communication between endothelial cells and sensory neurons using microfluidic devices by evaluating the mechanism of action and the signaling pathways involved in their crosstalk. We investigated the effect of SNs on ECs’ Ca^2+^ signaling, Cx43 expression and the impact on the NO endothelial-dependent pathways. We focused on the classical neuropeptides CGRP and SP, using their specific antagonists, as mechanism of action of SNs on ECs, evaluating also their respective role on this neurovascular coupling and cell signaling.

## Materials and methods

### Microfluidic devices fabrication

Microfluidic devices molds were obtained using standard photolithography and soft lithography procedures. Dimension measurements of the molds were performed through interferometry using an S neox 3D Optical Profiler confocal microscope (Sensofar Metrology). The devices were made with polydimethylsiloxane (PDMS) (Sylgard 184, Sigma-Aldrich, ref 761036) and polymerized at 60 °C for 3 h. Before use, devices were incubated for 4 days in absolute ethanol, dried and then exposed to UV light (254 nm) for 40 min. Microfluidic devices characterization was described in our previous work [3].

### Cell isolation and culture

Rat (*Rattus norvergicus*) neurons and endothelial cells were used. Primary sensory neurons (SNs) were harvested from dorsal root ganglia (DRGs) from 5 to 8 weeks old female Wistar rats, as described by Malin and colleagues [21] and in our previous work [3]. Briefly, spinal columns were removed and opened from the caudal to the rostral region in order to expose the DRGs. The DRGs were individually harvested and digested with 2800 U/mL of Collagenase Type IV (Gibco) for 1 h at 37 °C. Digestion products were then washed twice with Dulbecco’s Modified Eagle’s Medium 1 g/L glucose (DMEM, Gibco) and mechanically dissociated using fire-polished glass Pasteur pipettes. The cell suspension was finally washed three times with culture medium, and resuspended in DMEM 4.5 g/L glucose supplemented with 2% (v/v) B-27 Serum-Free Supplement (B-27, Gibco), 1% (v/v) fetal bovine serum (FBS, PAN Biotech) and 1% (v/v) Penicillin Streptomycin (Pen/Strep, Gibco). For nitric oxide experiments, phenol red-free DMEM 4.5 g/L glucose (Gibco) was used, supplemented as described previously.

Rat primary bone marrow-derived microvascular endothelial cells (ECs) were purchased from Cell Biologics (catalog # RA-6221). These cells are isolated from tibia and femur of 10 weeks old female Sprague-Dawley rats and cultured with endothelial cell growth medium-2MV (EGM-2MV; Lonza) at 37 °C in humidified atmosphere with 5% CO_2_. Cells were seeded at 10^4^ cells/cm^2^ and were used between passages 5 to 8. Calcitonin gene related peptide (CGRP) and substance P (SP) (Interchim, ref RP1109560.5 and RP10178 respectively) were added directly in the medium at concentrations of 100 nM for CGRP and 1 µM for SP. Culture media were changed every two days.

For co-culture assays, microfluidic devices were electrostatically attached over glass coverslips previously coated with 0.1 mg/mL Poly-D-Lysine (PDL, Sigma-Aldrich) and 20 µg/mL laminin (Sigma-Aldrich). ECs were seeded in the lateral compartments at a density of 80 cells/mm². SNs were seeded in the central compartment immediately after isolation at a density of 160 cells/mm². Fresh medium was added every two days and the cells were maintained in culture until day 7.

The antagonists BIBN4096 (for CGRP - AntCGRP) and SR140333 (for SP - AntSP) (Tocris, Bio-Techne) were solubilized in dimethyl sulfoxide (DMSO) considered as vehicle and added directly in ECs culture medium at a concentration of 10 µM each [22–24]. Vehicle final concentration was 0.2% v/v for all groups. In the experiments aiming nitric oxide quantification, EGM-2MV was replaced by phenol red-free EGM-2MV (Lonza) with 5% (v/v) FBS.

The co-culture of ECs and SNs in microfluidic devices was assessed by confocal microscopy at day 7. Briefly, cells were fixed with 2% (w/v) paraformaldehyde for 30 min at room temperature (RT), permeabilized with 0.1% (v/v) Triton X-100 for 5 min at RT and blocked with 1% (w/v) bovine serum albumin (BSA, GE Healthcare) for 30 min at RT. For SNs, primary rabbit anti-rat β-III tubulin antibody (Abcam, ab18207) was used at a 1:500 dilution at 4 °C overnight. Secondary goat anti-rabbit IgG conjugated with Alexa Fluor 568 (Invitrogen™, catalog #A11036) was used at a 1:400 dilution for 45 min at RT. ECs was labelled against von Willebrand factor (Abcam, ab195028) diluted at 1:100. Nuclei were stained with DAPI (4′, 6′-diamidino-2-phenylindole, Life Technologies™) at 1 µg/mL. Images were acquired in a Leica TCS SPE 5 Confocal Laser Scanning Microscope.

### Antagonist cytotoxicity

Cytotoxicity of BIBN4096 and SR140333 was assessed according to the international standard ISO10993-5. Briefly, ECs were seeded at 10,000 cells/cm^2^ and cultured in 96 well plates during 24 h with BIBN4096 or SR140333 from 0.1 nM to 10 µM. Wells containing 0.1% (v/v) Triton X100 were used as control of toxicity. Cell viability was assessed by the Neutral Red assay and cell metabolic activity by using an MTT assay and Resazurin reduction [25]. For neutral red uptake, neutral red (Sigma-Aldrich, ref N4638) was dissolved at 1.25% (w/v) in EGM-2MV without serum, and 100 µL of the prepared solution was added per well. After 3 h of incubation at 37 °C, the supernatant was replaced by 100 µL of a mixture (50:50) containing 50% (v/v) ethanol / 1% (v/v) acetic acid in water. For MTT assay, MTT (3-(4, 5-dimethylthiazolyl-2)-2, 5-diphenyltetrazolium bromide, Sigma-Aldrich, ref M2128) (1 mg/mL stock in EGM-2MV without serum and without phenol red) was added in 125 µL per well. After 3 h of incubation at 37 °C in 5% CO_2_, the supernatant was removed and formed formazan crystals were dissolved by adding 100 µL of dimethyl sulfoxide (DMSO, ref D5879, Sigma-Aldrich, France). In both cases, color intensity was quantified by measuring the absorbance at 540 nm using a spectrophotometer (Perkin Elmer, 2030 Multilabel Reader VictorTMX3). For resazurin assay, 150 µL of culture medium containing resazurin (0.01 mg/mL) was added to each well and the microplate was incubated for 3 h. Subsequently, 100 µL of supernatant was transferred to another 96 wells microplate and fluorescence was measured (exc = 530 nm, em = 590 nm, Victor X3, Perkin Elmer). The mean values of absorbance measurements obtained from colorimetric tests and their corresponding standard deviation were calculated from 6 repeats. The results are expressed as a percentage of EGM-2MV.

### Real-time Ca^2+^ fluorescence imaging

An analog of Fura 2, the Fura-PE3 AM (Interchim) was used for measuring real-time variations of intracellular Ca^2+^ in ECs. Cells were incubated in EGM-2MV medium (Lonza) with 5 µM Fura-PE3 AM at 37 °C and 5% CO_2_ for 30 min. Cells were washed with fresh medium and SNs in the central compartment were stimulated using capsaicin (Sigma-Aldrich) at a final concentration of 100 nM. In control microfluidic devices containing ECs cultured in absence of SNs, capsaicin was also added at 100 nM to the central compartment. Fura-PE3 AM was excited alternately at 340 and 380 nm, the emitted fluorescence was monitored at 510 nm. One image per second was acquired during 10 min. Fluorescence was observed using an epifluorescence microscope Olympus IX70 of random regions from the lateral compartment of a microfluidic device. Inside a region, we identified at least 8–14 ECs, in which a cell represents a region of interest (ROI). Metafluor software (Universal Imaging) was used to manually designate each cell as a ROI and measure the fluorescence intensity in each ROI. Intracellular calcium level was expressed as the ratio of the fluorescence intensity due to the excitation wavelengths 340 nm to 380 nm (Ratio (340/380 nm)). The delta variation in calcium level was calculated as the difference between the maximum value of the 340/380 nm ratio measured in ECs after capsaicin was added in the central compartment and value of the 340/380 nm ratio in ECs at the moment of capsaicin supplementation.

### RNA extraction, cDNA synthesis and RT-qPCR analysis

Total RNA was extracted from ECs using the RNeasy Plus Micro Kit (Qiagen) according to the manufacturer’s protocol. RNA concentration and purity (OD 260/280) were determined using a NanoPhotometer P330 (Implen GmbH). 100 to 500 ng of total RNA were reverse transcribed into cDNA using the Maxima Reverse Transcriptase kit (Thermo Scientific™), according to the manufacturer’s protocol. RT-qPCR experiments were performed using a Takyon™ No Rox SYBR 2X MasterMix dTTP blue (Kaneka Eurogentec S.A.) with standard PCR conditions for SYBR Green detection in a CFX Connect™ Real-Time PCR Detection System (Bio-Rad Laboratories) and analyzed with the CFX Manager™ software, version 3.0 (Bio-Rad Laboratories). Primer sequences are presented in Table 1. Target gene expression was quantified using the cycle threshold (Ct) values and relative mRNA expression levels were calculated according to the 2^− DDCt^ method. Ribosomal protein lateral stalk subunit P0 (*Rplp0*), glyceraldehyde 3-phosphate dehydrogenase (*Gapdh*) and hypoxanthine phosphoribosyl transferase 1 (*Hprt1*) were used as reference genes.

**Table 1:** Sequences of primers used to analyze ECs gene expression

Gene	Gene ID	Sequence	Fragment length (bp)
*Gja1* (Cx43)	24,392	F: 5’ AATAAATCATAATTCAATGGCTGCTCCTCACC 3’ R: 5’ AATAAATCATAATGTAGTTCGCCCAGTTTTGC 3’	139
*Rplp0*	64,205	F: 5’ CACTGGCTGAAAAGGTCAAGG 3’ R: 5’ GTGTGAGGGGCTTAGTCGAA 3’	187
*Gapdh*	24,383	F: 5’ GGCATTGCTCTCAATGACAA 3’ R: 5’ TGTGAGGGAGATGCTCAGTG 3’	213
*Hprt1*	24,465	F: 5’ AGCCTAAAAGACAGCGGCAA 3’ R: 5’ GGCCACAGGACTAGAACGTC 3’	87

### Protein extraction and Western blotting analysis

ECs were lysed directly in the microfluidic devices with RIPA buffer (Sigma), containing a Protease Inhibitor Cocktail (PIC, Sigma) and Halt™ Phosphatase (Thermo Scientific). Total protein content was quantified using Bradford Reagent (Sigma). For each sample 40 µg of total protein was denatured in loading buffer containing 1% (v/v) β-mercaptoethanol at 95 °C for 5 min, separated by sodium dodecyl sulphate-polyacrylamide gel electrophoresis (SDS-PAGE) on a 7.5% (v/v) polyacrylamide gel and electroblotted to the PVDF membrane (Immobiolon, Millipore). Membranes were blocked for 1 h with 5% (w/v) BSA (GE Healthcare) in a Tris-buffered saline (TBS) 0.1% Tween-20 (Sigma) solution. Membranes were immunoblotted overnight at 4 °C with primary antibodies diluted in TBS-Tween / BSA 5%. Antibodies and their dilutions are presented in Table 2. Immunodetection was performed using Clarity™ Western ECL detection kit reagents (Bio-Rad Laboratories). Membranes were visualized on ImageQuantTM LAS 4000 mini (GE Healthcare Life Sciences) and bands intensity was quantified using the ImageJ software.

**Table 2:** Antibodies and dilutions used for Western blot analysis of ECs protein expression

Antibody anti-	Host species	Reference	Supplier	Dilution
**Cx43**	Rabbit	C6219	Sigma	1:5000
**eNOS**	Rabbit	32,027	Cell Signaling	1:1000
**p-eNOS (T495)**	Rabbit	9574 S	Cell Signaling	1:1000
**p38**	Rabbit	Sc-728	Santa Cruz	1:1000
**p-p38**	Rabbit	9211	Cell Signaling	1:1000
**Erk1/2**	Rabbit	4695	Cell Signaling	1:1000
**p-ERK1/2**	Rabbit	4370	Cell Signaling	1:2000
**Gapdh**	Rabbit	G9545	Sigma	1:5000
**Rabbit (+ HRP)**	Goat	4052-05	SouthernBiotech	1:5000

### Nitrite production

After 7 days of culture in phenol red-free EGM-2MV supplemented with 5% (v/v) FBS, ECs supernatants were harvested, then nitrite and nitrate production by ECs was determined using the Nitrate/Nitrite Fluorometric Assay Kit (Cayman Chemical) according to the manufacturer’s protocol. Briefly, first the supernatants were centrifuged for 5 min at 10 000 g. Nitrate contained in the medium were converted to nitrite using nitrate reductase. Then, DAN (2,3-diaminonaphtalene) was added as an acidic solution, followed by NaOH which enhances the detection of the fluorescent product 1(H)-naphtotriazole. The fluorescence was measured on a Victor X3 2030 Multilabel Reader (PerkinElmer) at an excitation wavelength of 355 nm and an emission wavelength of 460 nm. Final nitrite production was calculated related to the standard curve and normalized by total protein concentration.

### Statistical analysis

Data is expressed as mean values of at least 3 independent experiments ± standard deviation. The Software Prism 5.0 (GraphPad Software Inc.) was used to analyze differences between groups. Student’s t test or one-way ANOVA followed by Bonferroni’s or Tukey’s multiple comparison test as post hoc when necessary. A value of *p* < 0.05 is considered statistically significant.

## Results

### SNs can stimulate Ca^2+^ influx in ECs

**Fig. 1:**
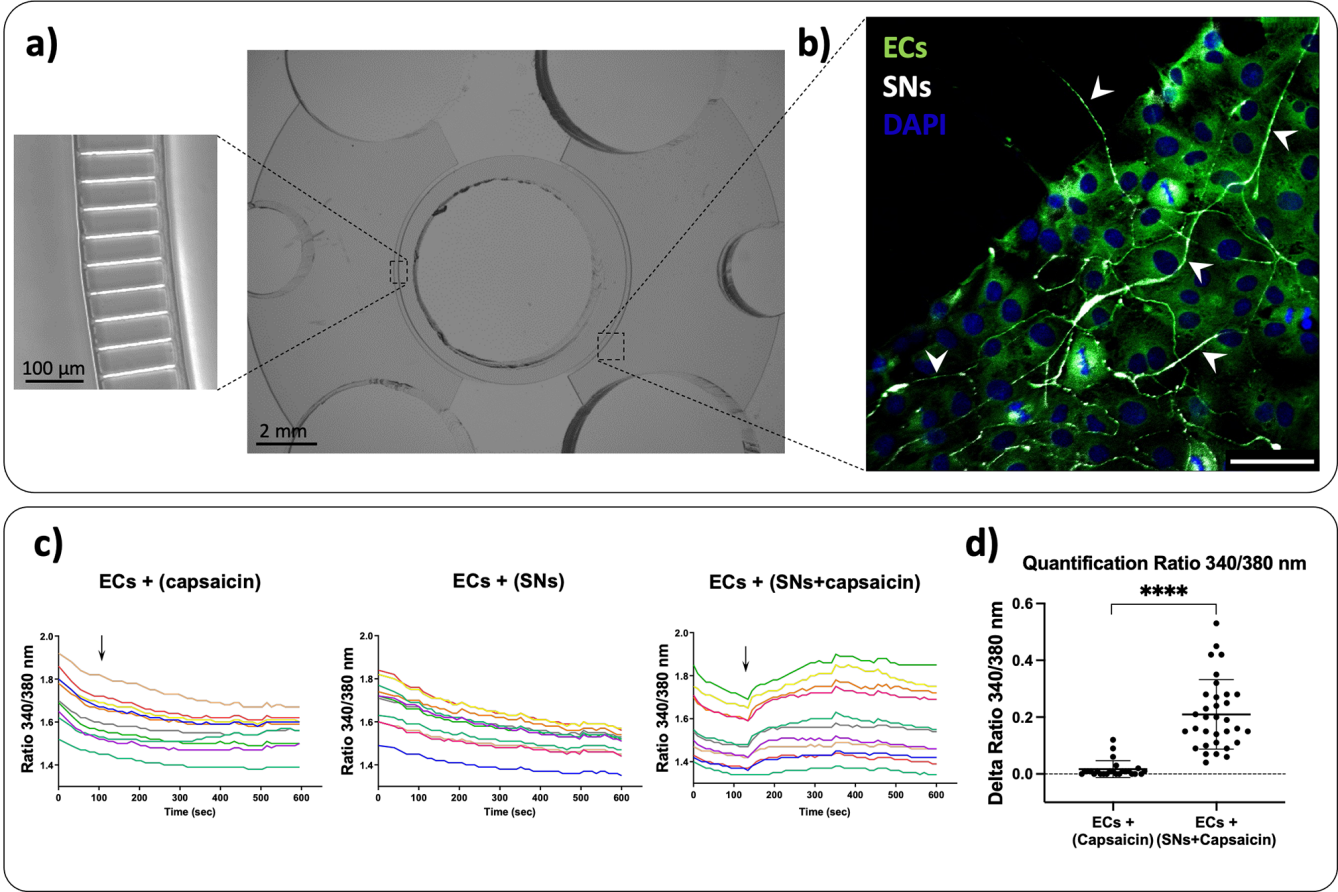
Representative images of the co-culture between ECs and SNs in microfluidic devices and intracellular Ca^2+^ levels in endothelial cells (ECs) in the presence or absence of sensory neurons (SNs). **(a)** Microfluidic devices composed of 3 compartments: one central compartment containing SNs surrounded by two lateral compartments containing ECs. Central and lateral compartments are separated by microchannels with precise dimensions that permit axons to pass through, as characterized in our previous study [3]. **(b)** Confocal microscopy representative image of the co-culture between SNs and ECs at day 7. SNs are shown in white (β-III tubulin) and ECs in green (von Willebrand factor). The white arrowheads show neurites emitted by the SNs passing through microchannels and within ECs culture. Scale bar = 50 μm. **(c)** Representative traces of a region of interest (ROI) around ECs from a random region in a microfluidic device showing the intracellular calcium levels in ECs cultured alone having capsaicin added in the central compartment (ECs + (capsaicin)), ECs culture in the presence of SNs without capsaicin stimulation (ECs + (SNs)) and ECs co-culture with SNs with capsaicin stimulation (ECs + (SNs + capsaicin)). Each line represents an endothelial cell previously defined as a ROI using the Metafluor software. The values of calcium levels are expressed as Ratio (340/380 nm) as a function of time. The black arrow represents the addition of capsaicin in the SNs compartment. These figures are representative of three independent experiments. **(d)** Variation of intracellular calcium levels in ECs quantified by Delta Ratio 340/380 nm as the maximum value of the 340/380 nm ratio measured in ECs after capsaicin addition in the central compartment minus the value of the 340/380 nm ratio in ECs at the moment of capsaicin addition. Data are represented as *n* = 24–42 ROIs from three independent experiments. *n* = 8–14 ROIs analyzed per microfluidic device. Mean values ± SD analyzed by Student’s t test, **** *p* < 0.0001

As previously described by our group, SNs emit neurites that pass through the microchannels towards ECs compartment where they interact closely (Fig. 1a-b). Our group has already described a direct effect of SNs on ECs functions in view of angiogenesis and extracellular matrix remodeling previously [3] using this same methodology. To evaluate if SNs impact on ECs is driven *via* the secretion of neuropeptides followed by a downstream Ca^+ 2^ signaling on ECs [12], we quantified the free intracellular Ca^2+^ ([Ca^2 +^ _i_]) in ECs. It is well established in the literature that capsaicin stimulates SNs leading to the release of extracellular vesicles containing among others, the classical sensory neuropeptides CGRP and SP [26, 27] that could activate calcium signaling.

Here, we cultured ECs alone or in the presence of SNs in microfluidic chambers for 7 days and we stimulated SNs or not, with capsaicin. When SNs are not stimulated with capsaicin, or when ECs were cultured alone and capsaicin is added in the culture medium in the central compartment deprived of SNs, no effect is observed on the basal calcium levels. However, when ECs were co-cultured with SNs and latter stimulated with capsaicin, a synchronized increase in calcium level is observed in the ECs (Fig. 1c), revealing an increase in the calcium level within 2 s, then the calcium intensity signal reached a plateau for all cells, which was maintained for at least 10 min. These findings were confirmed when we quantified the variation of intracellular calcium levels using the delta Ratio 340/380 nm (Fig. 1d).

### Cx43 expression and phosphorylation are not affected in ECs co-cultured with SNs

**Fig. 2:**
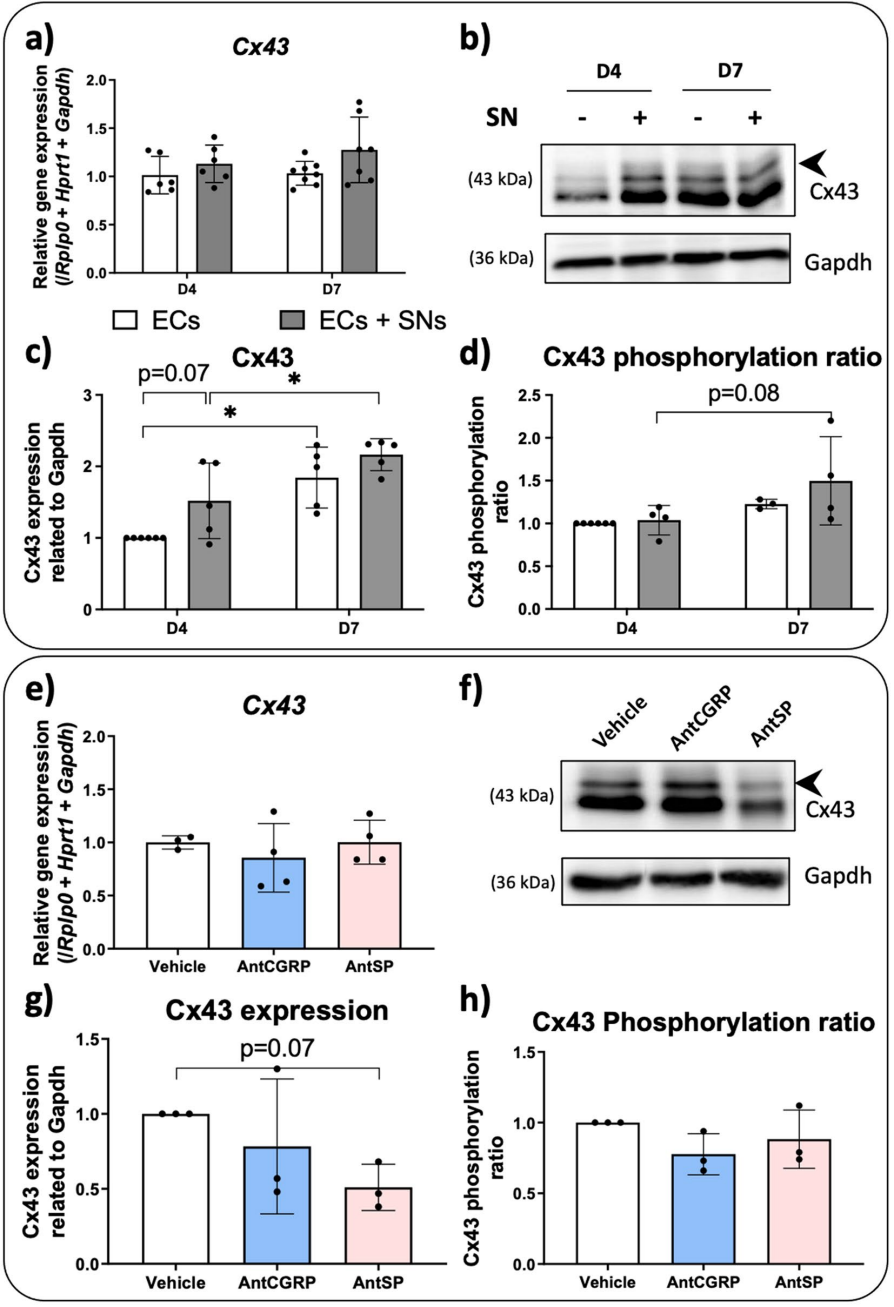
Effect of (**a**-**d**) the co-culture of ECs with SNs and (**e**-**h**) CGRP and SP antagonists on Cx43 expression and phosphorylation in endothelial cells (ECs). **(a)** Gene expression of *Cx43* by ECs cultured in presence (grey bars) or absence (white bars) of SNs after 4 and 7 days. **(b)** Representative western blot of Cx43 protein expression by ECs cultured in presence (+) and absence (-) of SNs after 4 and 7 days of culture. Black arrowheads represent phosphorylated forms of Cx43 (pCx43) with slightly higher molecular weight. **(c)** Total Cx43 protein expressed by ECs cultured in presence (grey bars) or absence (white bars) of SNs after 4 and 7 days relative to EC alone Gapdh at day 4. **(d)** Phosphorylated and non-phosphorylated forms of Cx43 were quantified separately and the Cx43 phosphorylated ratio (pCx43/Cx43) was evaluated relative to ECs alone at day 4. (**a**-**d**) *n* = 6 independent experiments. **(e) ***Cx43* gene expression in the presence of AntCGRP, AntSP and vehicle after 7 days of co-culture between ECs and SNs in the presence of each antagonist and vehicle. **(f)** Representative western blot of Cx43 protein expression relative to Gapdh after 7 days of co-culture between ECs and SNs in the presence of each antagonist and vehicle. Black arrowheads represent pCx43 form. **(g)** Total Cx43 protein expression relative to Gapdh in ECs co-cultured with SNs for 7 days in the presence of each antagonist relative to the vehicle. **(h)** Phosphorylated and non-phosphorylated forms of Cx43 in ECs cultured with SNs for 7 days in the presence of each antagonist and vehicle. The Cx43 phosphorylated ratio (pCx43/Cx43) was quantified relative to the vehicle. (**e**-**h**) *n* = 3 independent experiments. All graphs represent mean values ± SD for both gene and protein evaluation using ANOVA test followed by Bonferroni (**a**, **c** and **d**) or Tukey (**e**, **g** and **h**) as post-hoc, * *p* < 0.05. Uncropped western blots images are shown in Supplementary Fig. 2

It is well known that calcium propagation to neighboring cells is also mediated through gap junctions and Cx43 hemichannels [28, 29], and Cx43 is highly expressed in ECs [30]. Moreover, considering the synchronized calcium response in ECs after SNs stimulation with capsaicin (Fig. 1c-d), we wonder if SNs and neuropeptides CGRP and SP could affect the expression of Cx43, possibly affecting then the calcium propagation. We evaluated the gene and protein expression of Cx43 in ECs cultured in the presence or absence of SNs after 4 and 7 days of culture. Firstly, when we analyzed the *Cx43* gene expression (Fig. 2a), no significant difference was observed, in both time points. We then analyzed Cx43 protein expression by Western blot (Fig. 2b, Supplementary Fig. 2) followed by densitometric analysis and using Gapdh as reference protein for quantification purposes (Fig. 2c). At day 4, there is a tendency of upregulation of Cx43 protein expression when ECs are co-cultured with SNs relative to ECs cultured alone (*p* = 0.07). The expression of Cx43 increased over time in ECs alone or in the presence of SNs (Fig. 2c).

In order to evaluate Cx43 regulation by phosphorylation, we analyzed the phosphorylated forms by western blot [31] as indicated by the black arrowhead in Fig. 2b. When the phosphorylated forms of Cx43 (pCx43) were analyzed and the Cx43 phosphorylation ratio (pCx43/Cx43) was calculated, a tendency in the upregulation of pCx43 from day 4 to day 7 is observed when the ECs are cultured in the presence of SNs (*p* = 0.08) (Fig. 2d).

To further explore the underlying mechanism of action in which SNs can modulate ECs activity, and based on our previous work [3] that demonstrates that CGRP and SP produced by SNs are both involved in the upregulation of the angiogenic markers, the two sensory neuropeptide antagonists BIBN4096BS (AntCGRP) and SR140333 (AntSP) were supplemented in ECs culture medium for 7 days in the presence of SNs. The control used to quantify the *Cx43* gene expression (Fig. 2e), as well as the protein and Cx43 phosphorylation ratio (Fig. 2f, Supplementary Fig. 2), was the vehicle used to solubilize both antagonists (DMSO at 0.2% v/v final concentration for all groups). At genetic level, when the co-culture between ECs and SNs is supplemented separately with AntCGRP or AntSP, no significant difference was observed relative to the vehicle (Fig. 2e) after 7 days of co-culture of ECs with SNs. When the Cx43 protein expression by ECs is analyzed by western blot (Fig. 2f), a tendency of downregulation is observed in the presence of AntSP (*p* = 0.07) (Fig. 2g) compared to the control (vehicle alone). However, when the Cx43 phosphorylation ratio is evaluated, no difference was observed between groups (Fig. 2h).

### NO production increased in ECs co-cultured with SNs: CGRP as regulator

**Fig. 3:**
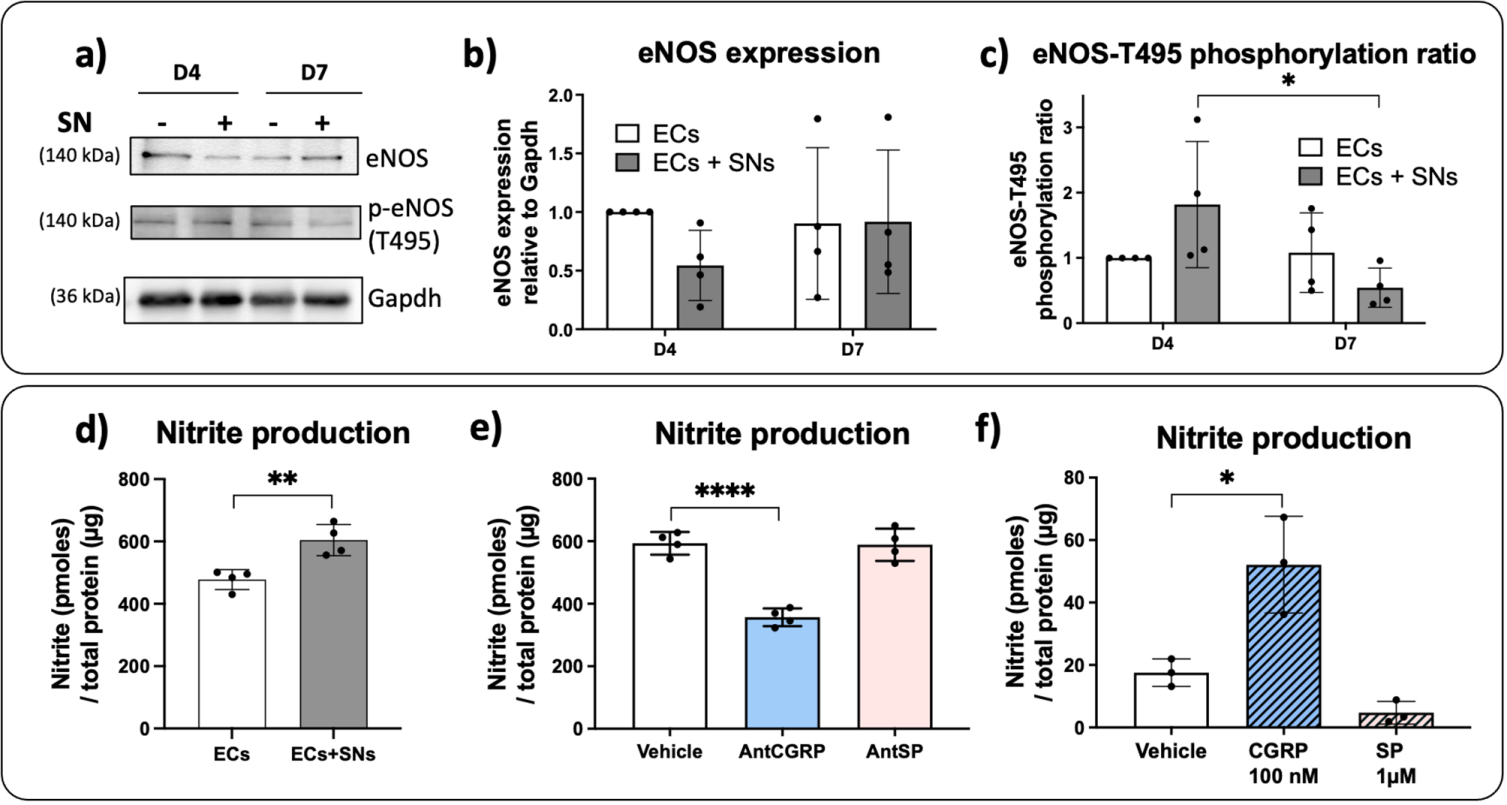
Effect of (**a**-**c**) the co-culture of ECs with SNs and (**d**-**f**) CGRP and SP on eNOS expression and nitrite production. **(a)** Representative western blot of eNOS protein expression by ECs cultured in presence (+) and absence (-) of SNs after 4 and 7 days of culture. The phosphorylation of T495 site was assessed using specific antibody (p-eNOS) (Table 2). **(b)** eNOS protein expression relative to Gapdh in ECs cultured in presence (grey bars) or absence (white bars) of SNs relative to ECs alone at day 4. **(c)** Phosphorylation ratio of the T495 site (p-eNOS/eNOS) relative to ECs alone at day 4. **(d)** Quantification of NO degradation products was performed at day 7 in the ECs culture medium in the presence or absence of SNs. **(e)** Quantification of NO degradation products was performed at day 7 in the ECs culture medium supplemented with CGRP and SP antagonists in the presence or absence of SNs. The vehicle group is considered as control. **(f)** Effect of the two neuropeptides CGRP (100 nM) and SP (1 µM) separately on nitrite production by ECs cultured alone for 7 days. ECs medium supplemented with the vehicle is considered as control (*n* = 4 independent experiments). The graphs represent mean values ± SD using ANOVA test followed by Bonferroni (**b**, **c**) or Tukey (**e**, **f**) as post-hoc or Student’s t test (**d**), * *p* < 0.05, ** *p* < 0.01, **** *p* < 0.0001. Uncropped western blots images are shown in Supplementary Fig. 3

Increases of [Ca^2 +^ _i_] in ECs can be also linked to an augmentation of nitric oxide (NO) production through the regulation of endothelial NO synthase (eNOS) expression or activity [32]. Thus, the endothelial marker eNOS was evaluated in ECs co-cultured with SNs after 4 and 7 days. We analyzed by western blot the eNOS protein expression as well as the specific phosphorylation site T495 (p-eNOS) (Fig. 3a, Supplementary Fig. 3), which is considered to be inhibitory in NO production [33]. In the presence of SNs, no significant difference was observed on the eNOS expression by ECs after 4 and 7 days of co-culture related to ECs cultured alone (Fig. 3b). However, when the phosphorylation ratio of T495 site was evaluated, a significant downregulation of the T495 phosphorylation site is observed in ECs cultured the presence of SNs over time, from day 4 to day 7 (Fig. 3c).

In parallel, we measured the NO production through the specific degradation products of NO in the ECs culture medium at day 7 for both groups of cultures. Interestingly, although absence of statistical difference on eNOS protein expression and phosphorylation ratio in the presence *versus* absence of SNs, a significantly higher nitrite concentration is observed when ECs are co-cultured with SNs relative to ECs cultured alone after 7 days of culture (Fig. 3d).

Again, to evaluate the role of the two sensory neuropeptides CGRP and SP on the NO production, we then measured the nitrite production in the co-culture of ECs and SNs in the presence of AntCGRP or AntSP (Fig. 3e). We observed that nitrite concentration was significantly decreased in the presence of AntCGRP (*p* < 0.0001), but no significant difference was observed after AntSP treatment (Fig. 3e). In order to confirm the effect of CGRP on NO production, we then cultured ECs alone in the presence of the two neuropeptides CGRP or SP added in the culture medium separately, at 100 nM and 1 µM, respectively. These concentrations were chosen as they do not induce cytotoxicity (Supplementary Fig. 1) and according to our previous work, as they showed an effect on ECs gene expression of *VegfA* and *Mmp2*, respectively [3]. When ECs were treated with 100 nM CGRP, a significantly higher production of nitrite was observed relative to the vehicle alone (*p* < 0.05) (Fig. 3f). No significant difference was detected in nitrite production when ECs were treated with 1 µM SP compared to the vehicle.

### Mitogen-activated protein kinases (MAPK) expression in ECs co-cultured with SNs: Erk1/2 regulated by SP?

MAP kinases activation such as p38 and Erk1/2 can result in downstream eNOS regulation activity through its phosphorylation sites, thus participating in the angiogenesis process [34–36]. Then, we evaluated the p38 and Erk1/2 pathways in ECs co-cultured with SNs for 4 and 7 days.

**Fig. 4:**
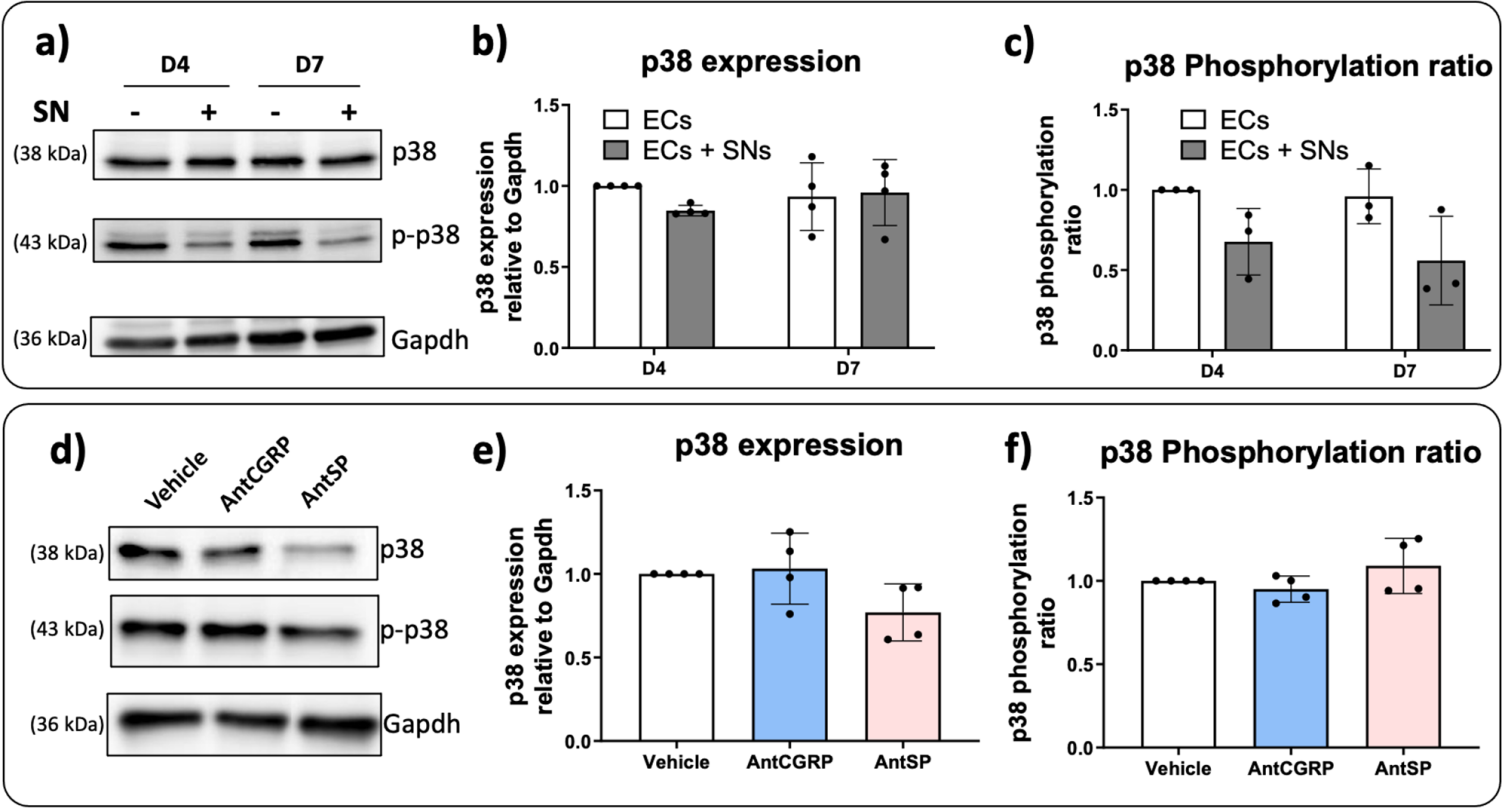
Effect of (**a**-**c**) the co-culture of ECs with SNs and (**d**-**f**) CGRP and SP antagonists on p38 expression. **(a)** Representative western blot of p38 and its phosphorylated form (p-p38) expression by ECs cultured in presence (+) and absence (-) of SNs after 4 and 7 days of culture. P-p38 expression was assessed using specific antibody (Table 2). **(b)** p38 expression relative to Gapdh by ECs cultured in presence (grey bars) or absence (white bars) of SNs, after 4 and 7 days relative to ECs alone at day 4. **(c)** Phosphorylated and non-phosphorylated forms of p38 were quantified separately and the phosphorylated ratio (p-p38/p38) was evaluated relative to ECs alone at day 4. **(d)** Representative western blot of p38 and p-p38 expression in EC when co-cultured with SNs for 7 days in the presence of CGRP and SP antagonists (AntCGRP and AntSP, respectively). The vehicle group is used as control. **(e)** Total p38 expression relative to Gapdh in ECs co-cultured with SNs for 7 days in the presence of each antagonist relative to the vehicle. **(f)** Phosphorylation ratio (p-p38/p38) in the presence of each antagonist relative to the vehicle (*n* = 4 independent experiments). The graphs represent mean values ± SD using ANOVA test followed by Bonferroni (**b**, **c**) or Tukey (**e**, **f**) as post-hoc. Uncropped western blots images are shown in Supplementary Fig. 4

Firstly, we evaluated the expression of p38 and its phosphorylated form (p-p38) by western blot (Fig. 4a, Supplementary Fig. 4). No significant differences were observed in the p38 expression as well as in the phosphorylation ratio when ECs were co-cultured with SNs after 4 and 7 days (Fig. 4b-c). Nevertheless, we further evaluated the effect of AntCGRP and AntSP in the ECs culture medium in the presence of SNs after 7 days. We analyzed p38 and p-p38 expression by western blot (Fig. 4d, Supplementary Fig. 4) and evaluated the protein expression (Fig. 4e) as well as the phosphorylation ratio (Fig. 4f). No significant differences were found between AntCGRP or AntSP treatment relative to the vehicle group for p38 expression nor for the phosphorylation ratio.

**Fig. 5:**
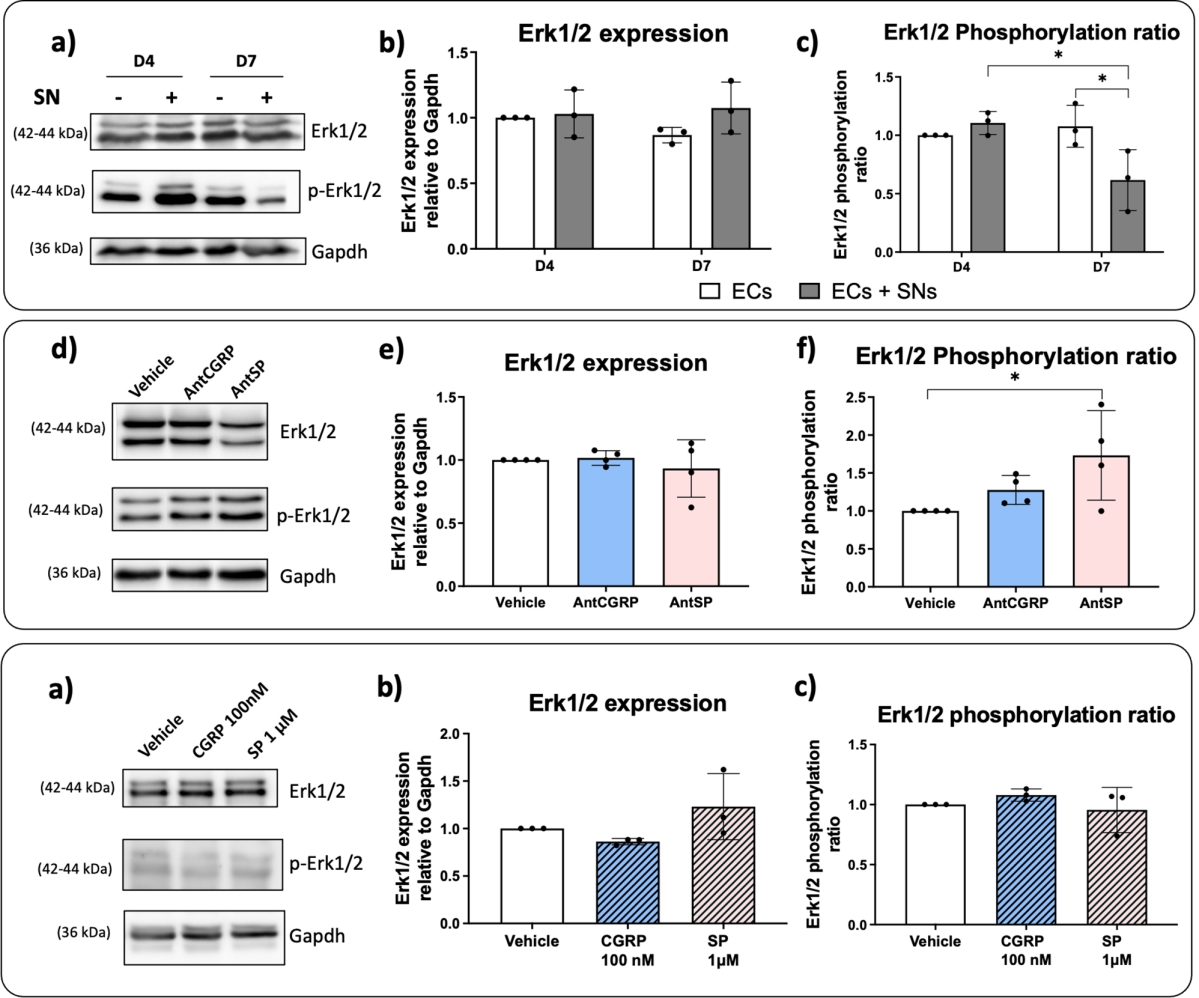
Effect of (**a**-**c**) the co-culture of ECs with SNs, (**d**-**f**) CGRP and SP antagonists, and (**g**-**i**) the neuropeptides CGRP and SP on Erk1/2 expression and phosphorylation. **(a)** Representative western blot of Erk1/2 and its phosphorylated form (p-Erk1/2) expression by ECs cultured in presence (+) and absence (-) of SNs after 4 and 7 days of culture. P-Erk1/2 expression was assessed using specific antibody (Table 2). **(b)** Erk1/2 expression relative to Gapdh by ECs cultured in presence (grey bars) or absence (white bars) of SNs, after 4 and 7 days relative to ECs alone at day 4. **(c)** Phosphorylated and non-phosphorylated forms of Erk1/2 were quantified separately and the phosphorylated ratio (p-Erk1/2-to-Erk1/2) was evaluated relative to ECs alone at day 4. (**a**-**c**) *n* = 4 independent experiments. **(d)** Representative western blot of Erk1/2 and p-Erk1/2 expression in presence of CGRP or SP antagonists (AntCGRP and AntSP, respectively) and vehicle. **(e)** Erk1/2 expression relative to Gapdh by ECs co-cultured with SNs in the presence of each antagonist relative to the vehicle. **(f)** Erk1/2 phosphorylation ratio (p-Erk1/2-to-Erk1/2) in the presence of each antagonist relative to the vehicle. (**d**-**f**) *n* = 3 independent experiments. **(g)** Representative western blot of Erk1/2 and p-Erk1/2 expression in ECs cultured in well plates in the presence of 100 nM CGRP, 1 µM SP, and vehicle. **(h)** Erk1/2 expression relative to Gapdh in ECs alone cultured in the presence of 100 nM CGRP, 1 µM SP relative to the vehicle. **(i)** Erk1/2 phosphorylation ratio in the presence of 100 nM CGRP, 1 µM SP relative to the vehicle. (**g**-**i**) *n* = 3 independent experiments. The graphs represent mean values ± SD using ANOVA test followed by Bonferroni (**b**, **c**) or Tukey (**e**, **f**) as post-hoc, * *p* < 0.05. Uncropped western blots images are shown in Supplementary Fig. 5

Then, Erk1/2 expression, its phosphorylated form (p-Erk1/2) and the phosphorylation ratio were also evaluated by western blot (Fig. 5a, Supplementary Fig. 5). When ECs were co-cultured with SNs for 4 and 7 days, no significant differences in the Erk1/2 expression were observed (Fig. 5b). When we analyzed the phosphorylation ratio, no difference was observed at day 4. However, at day 7 the p-Erk1/2 form was significantly downregulated in the presence of SNs relative to day 4, and also significantly downregulated relative to the ECs cultured alone (Fig. 5c).

Again, we tested the effect of AntCGRP and AntSP on the protein expression of both Erk1/2 and p-Erk1/2 by western blot (Fig. 5d, Supplementary Fig. 5) when ECs were co-cultured with SNs for 7 days. No difference on the Erk1/2 expression was observed in the presence of AntCGRP or AntSP relative to the vehicle (Fig. 5e). No significant difference is observed in the phosphorylation ratio when AntCGRP is used. However, there is a significant upregulation of p-Erk1/2 in the presence of AntSP relative to the vehicle (Fig. 5f).

In order to explore a possible role of SP on Erk1/2 expression and phosphorylation, we cultured ECs in well plates in the presence of 100 nM CGRP and 1 µM SP and analyzed by western blot (Fig. 5g, Supplementary Fig. 5). No significant differences were observed in Erk1/2 expression (Fig. 5h) and in the Erk1/2 phosphorylation ratio (Fig. 5i) in relation to the vehicle.

## Discussion

Neurovascular coupling is a set of mechanisms regulating the exchanges between nerves and blood vessels, and more precisely at the cellular level, between endothelial cells and neural cells. Some of these mechanisms have been described [37, 38], but most of them remain unclear, which confirm the need for further study of the interplay between these cell types that can support the regulation of angiogenesis by nerve fibers. In more details, through this coupling, local neuronal activity plays an important role on angiogenesis and physiology of neighboring microvessels. In this context and based on our previous data obtained in vitro on the effect of sensory neurons on endothelial cells [3], our aim here was to discriminate the cell signaling involved in the neurovascular coupling and to focus on the role of the two neurotransmitters CGRP and SP produced by sensory neurons on the cell communication with endothelial cells.

Among the responses of endothelial cells to different chemical (e.g., neurotransmitters) and physical stimuli (e.g., shear stress pulsatile stretch), the increase in intracellular Ca^2+^ concentration can further trigger diverse signaling pathways, known to regulate the expression of angiogenic markers and EC functions [39].

In this work, we showed that the stimulation of SNs with capsaicin results an increase on intracellular calcium level in ECs (Fig. 1c-d). The effect of stimulation of SNs by capsaicin was observed on a group of endothelial cells whose resting [Ca^2 +^ _i_] had been measured. This observation suggests that the stimulation of SNs by capsaicin and the subsequent release of vesicles [40], including CGRP and SP, can be responsible for the calcium influx observed in ECs. The role of CGRP and SP has been previously identified in our work as mediators stimulating the gene expression of angiogenic markers of endothelial cell functions [3]. Our results also suggest that [Ca^2 +^ _i_] is increased in ECs in response to SNs stimulation with capsaicin and that ECs communicate quickly to present a harmonized response.

In particular, calcium signaling is known to occur *via* gap junctions and connexin (Cx) hemichannels [30]. Gap junctions are constituted by Cx that contain multiple phosphorylation sites and the phosphorylation state is well known to regulate the opening or closing of channels [41]. Among the Cx, Cx43 is one of the most widely expressed connexin in tissues and cell lines, especially in the vascularized bone tissue [41]. Phosphorylation of Cx43 is known to be involved in the regulation of hemichannels and gap junctional communication through trafficking, assembly/disassembly, degradation as well as gap junction channel gating [42]. More precisely, it has been described that Cx43 phosphorylation decreases intercellular communication [42–45], however, Ca^2+^ transfer is rarely affected. Indeed, even if Cx43 phosphorylation can inhibit other molecules transfer such as IP3, it allows Ca^2+^ movement [46]. Thus, the calcium signaling in ECs observed in presence of SNs in the Fig. 1 might still be mediated through Cx43 activity.

When looking at Cx43 in our co-culture model of ECs with SNs, an increase in Cx43 expression over time likely due to the increase in culture confluence (Fig. 2c), and a tendency in the phosphorylation rate were observed over time in the presence of SNs (Fig. 2d). Moreover, Cx43 protein expression tends to be upregulated in the presence of SNs relative to ECs cultured alone at day 4. An upregulation of Cx43 has been shown to positively modulate the angiogenic potential of endothelial cells by stimulating endothelial cell migration and network formation [47]. However, here, antagonists of CGRP and SP had no significant influence on the Cx43 protein expression and phosphorylation ratio. Antagonist of SP may have showed a tendency to decrease protein expression (*p* = 0.07), but it did not influence significantly the phosphorylation ratio. These results should be interpreted with caution and should be confirmed by further experiments before concluding on the role of SNs and these neuropeptides to control site-specific connexin phosphorylation, associated with a control of gap junctional communication through Cx43.

NO is also a potent mediator produced by the eNOS in ECs and is involved in endothelial functions by maintaining vascular homeostasis for example [48]. A Ca^2+^-dependent regulation of eNOS producing NO has already been described [49]. The eNOS is an important signal generator involved in the control of vascular tone and angiogenesis, regulated by several kinases acting at different sites to activate or inhibit NO production. Our results show that SNs increase NO production in ECs, probably involving a decrease in the eNOS T495 phosphorylation site without affecting eNOS protein expression, resulting thus in a stimulatory effect on eNOS activity. While SP seems not to be involved in the increase of NO production (Fig. 3e-f), the neuropeptide CGRP seems to be one of the mediators of this communication, since NO production is decreased in the presence of AntCGRP in the co-culture of ECs and SNs and increased when ECs are stimulated with synthetic CGRP. Our data are in accordance with the well-known vasodilator role of CGRP [50] and importantly, identify an important mechanism in which SNs can regulate ECs functions, in a NO-dependent manner.

Interestingly, a study conducted by Gaete and coauthors have suggested a contradictory effect of CGRP on NO production in mesenteric arterial beds [22]. The authors showed that CGRP signaling inhibits NO production through gap junction protein pannexin-1 channel activation in ECs. Besides we did not evaluate the role of pannexin-1, here we used ECs isolated from bone marrow microvasculature. The differences in the cells and tissues origin as well as the presence of different cell types in mesenteric arterial beds may explain the contradictory results observed in both situations. We developed an in vitro model to elucidate the cell communication and signaling between dissociated Dorsal Root Ganglia (DRG) cultures (including a mix of SNs and Schwann cells) and endothelial cells. Our approach does not reflect the complexity of the vascular tissue composition and response to SNs stimulation, however it allows us to describe the particular ECs response and to identify the main actors of their communication with SNs here and in our previous study [3].

Recently, an interesting relation between NO, Cx43 and Ca^2+^ signaling has been suggested to regulate endothelial cell migration, proposed by Espinoza and Figueroa [51]. Using rat primary microvascular endothelial cells and a 2D migration scratch assay, the authors suggested the involvement of NO-mediated S-nitrosylation in Cx43 hemichannels’ functions [51]. The activation of Cx43 hemichannels by S-nitrosylation should play a critical role in the long-lasting increase in [Ca^2 +^ _i_] that directs endothelial cell migration and network formation, suggesting that this mechanism may contribute to the NO-mediated effects in angiogenesis. The data previously published by our team showing that SNs and the neuropeptides SP and CGRP participate in the upregulation of angiogenesis markers in ECs such as *Angpt1*, *Col4*, *Mmp2* and interestingly, *VegfA* [3]. As well known, VEGF play an important role in ECs by contributing to vascular integrity and endothelial homeostasis [52, 53] and but also it stimulates eNOS expression and activation [49, 54]. Here, we show that CGRP regulates NO production, likely indirectly participating in the activation of Cx43 channels through NO-mediated S-nitrosylation, permitting the Ca^2+^ entry and thus triggering multiple mechanisms stimulating ECs functions. In this sense, our findings presented here show the increase in [Ca^2 +^ _i_] in ECs after SNs stimulation with capsaicin, a significant upregulation of NO production in ECs co-cultured with SNs, and only a tendency in Cx43 protein expression in ECs co-cultured with SNs at day 4 (*p* = 0,07). These data are in accordance with Espinoza’s and Figueroa’s observations and confirm that SNs play an important role in the control of the endothelial cell functions such as migration and network formation.

MAPK signaling proteins such as ERK1/2 and p38 are also known to be involved in processes such as angiogenesis by regulation of eNOS [55, 56] for example. Moreover, the ERK1/2 and p38 have been shown to be activated by the recognition of SP and CGRP, respectively, by their receptors in mesenchymal stem cells [57]. This activation leads to the migration of MSCs to bone formation sites and increased osteogenesis. Here, p38 protein expression and p38 phosphorylation ratio are not modulated by the presence of SNs nor by the presence of AntCGRP and AntSP at day 4 and day 7. Regarding Erk1/2, a downregulation of phosphorylation ratio was observed in ECs co-cultured with SNs at day 7 relative to day 4 and ECs cultured alone at day 7 (Fig. 5c), followed by an upregulation of Erk1/2 phosphorylation ratio in the presence of AntSP at day 7 (Fig. 5f). However, the effect of the neuropeptide SP on Erk1/2 phosphorylation was not confirmed when ECs alone were cultured in well plates in the presence of 1µM SP. It is important to note that when we identified the downregulation of Erk1/2 phosphorylation ratio at day 7 and the upregulation of Erk1/2 phosphorylation ratio in the presence of AntSP, the ECs were co-cultured with SNs, reflecting a more complex physiological communication between both cell types relative to the culture of ECs alone with the culture medium supplemented with CGRP and SP individually. Indeed, the neuropeptides availability and concentrations are not the same for both situations, which can explain the absence of effect of SP on Erk1/2 phosphorylation ratio when SP was added to the ECs culture medium (Fig. 5i). It is known that SNs secrete other molecules and neuropeptides, which could take over or act in synergy with SP, explaining why SP in ECs culture medium does not significantly affect Erk1/2 phosphorylation ratio. A similar scenario was observed in our previous study [3], in which we identified that SP when in culture medium can upregulate *Mmp2* expression, however when ECs were co-cultured with SNs in the presence of AntSP, *Mmp2* expression was only significantly reduced when AntSP and AntCGRP were used combined, suggesting that other molecules might be involved in the regulation of *Mmp2* expression by SNs.

It is well described in the literature that in ECs, Erk1/2 phosphorylation increases as a quick response followed by an angiogenic signal, and then the phosphorylation level is decreased due to the activity of Erk1/2 phosphatases [58, 59]. Here, the time course used for the analysis of Erk1/2 expression and phosphorylation in the co-cultured ECs (4 and 7 days allowing the neurite outgrowth), that could impact angiogenesis, could not be appropriate with a signal of the MAPK activities. However, in our study, when we consider the Erk1/2 phosphorylation ratio in ECs co-cultured with SNs from day 4 to 7, we observe a decrease in the phosphorylation ratio. This downregulation could be the result of the Erk1/2 phosphatases activity, acting on a return of Erk1/2 phosphorylation ratio to a base level after an earlier stimulation. Moreover, EC culture medium is a growth factor-rich environment, including VEGF-A in the composition, which could explain the high levels of Erk1/2 phosphorylation ratio in the group of ECs cultured alone at both time points.

## Conclusion

**Fig. 6:**
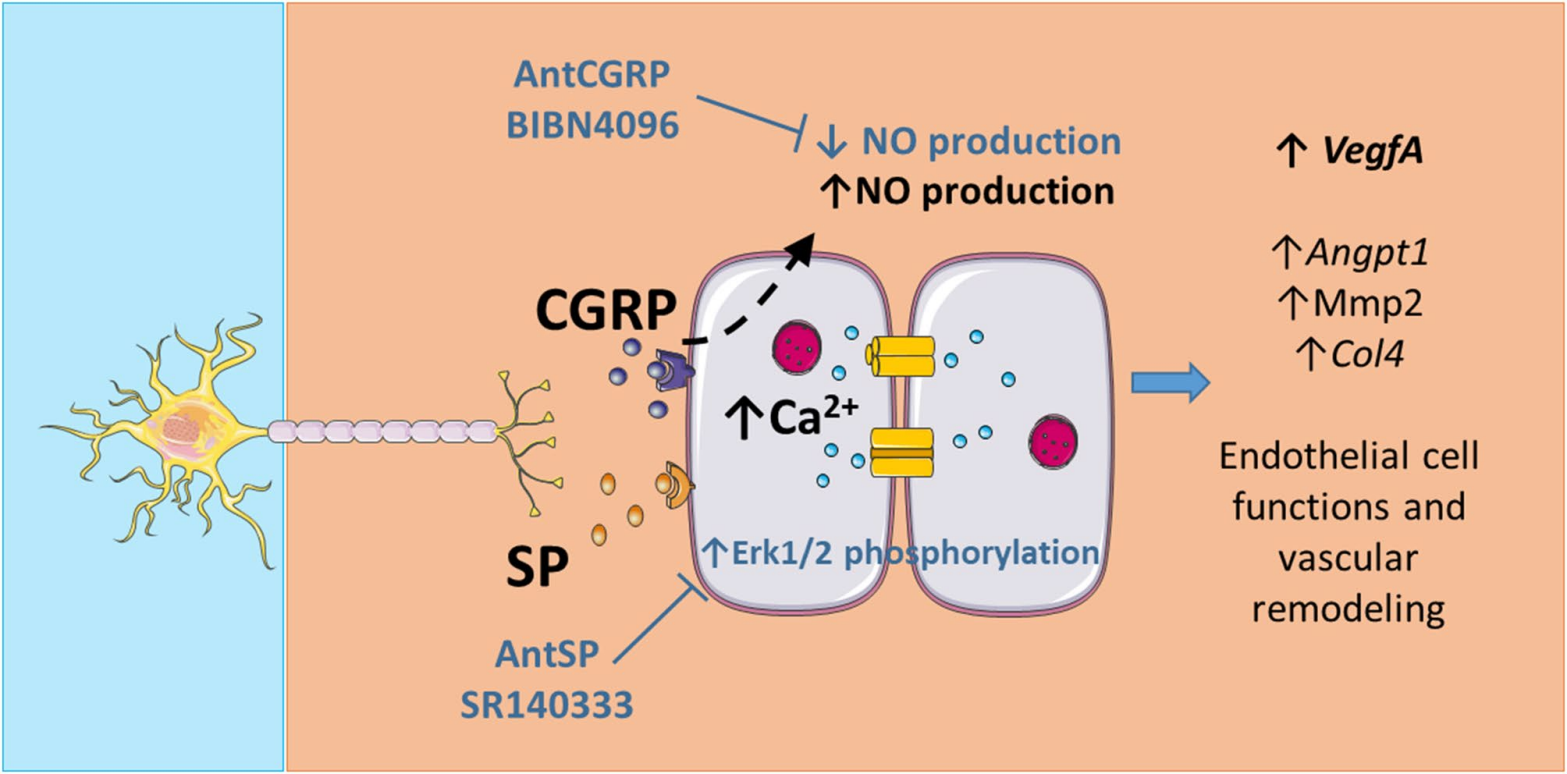
Putative role and mechanisms of action of sensory neurons (SN) on endothelial cells (ECs). Capsaicin-stimulated SNs induce Ca^2+^ influx in ECs. The presence of AntSP (SR140333) increases phosphorylation of Erk1/2 and a tendency on Cx43 downregulation (*p* = 0.07) is also observed. SNs induce an increase of NO production in ECs, and the neuropeptide CGRP seems to be the mediator of this effect since NO production is decreased in the presence of AntCGRP (BIBN4096) in the co-culture of ECs and SNs and increased when ECs are stimulated with synthetic CGRP. Considering our previous work showing that SNs regulates endothelial cell functions and vascular remodeling through the upregulation of *VegfA*, *Angpt1*, *Mmp2* and *Col4* [3] and the effect of VEGFA on NO production [14], SNs seem to play an important role in the control of ECs angiogenic functions

We showed that capsaicin-stimulated SNs could induce Ca^2+^ influx in ECs and we analyzed the effect of the two classical sensory peptides CGRP and SP on the protein levels and phosphorylation of potential actors impacted by the calcium influx in ECs. SNs increased NO production in ECs, probably involving a decrease in the inhibitory eNOS T495 phosphorylation site. The neuropeptide CGRP, produced by SNs, seems to be one of the mediators of this effect in ECs since NO production is decreased in the presence of AntCGRP in the co-culture of ECs and SNs, and increased when ECs are stimulated with synthetic CGRP. Considering this data and those in the literature, we propose that SNs play an important role in the control of the endothelial cell functions such as migration and network formation. A summary of our findings is presented in the Fig. 6.

## Electronic supplementary material

Below is the link to the electronic supplementary material.

Supplementary Material 1

## Data Availability

All data generated or analyzed during this study are included here.

## Abbreviations

ANGPT1: Angiopoietin 1
BDNF: Brain-derived neurotrophic factor
CGRP: Calcitonin gene-related peptide
COL4: Type 4 collagen
Cx: Connexin
DRG: Dorsal root ganglion
EC: Endothelial cell
ECM: Extracellular matrix
EGM-2MV: Microvascular Endothelial Cell Growth Medium-2
eNOS: Endothelial nitric oxide synthase
ERK1/2: Extracellular signal-regulated kinases 1/2
GMP: Guanosine monophosphate
MMP: Matrix metalloproteinase
MSC: Mesenchymal stem cell
NO: Nitric oxide
PECAM1: Platelet endothelial cell adhesion molecule 1
SP: Substance P
VEGF: Vascular endothelial growth factor
