# Supplementary material for: The effect of CGRP and SP and the cell signaling dialogue between sensory neurons and endothelial cells
Source: Biol Res. 2024 Sep 11;57:65. doi: 10.1186/s40659-024-00538-6 (PMC11389267; doi:10.1186/s40659-024-00538-6)
Supplement: Supplementary file 1 — Supplementary Material 1 [file 40659_2024_538_MOESM1_ESM.docx]

**Supplementary data :**


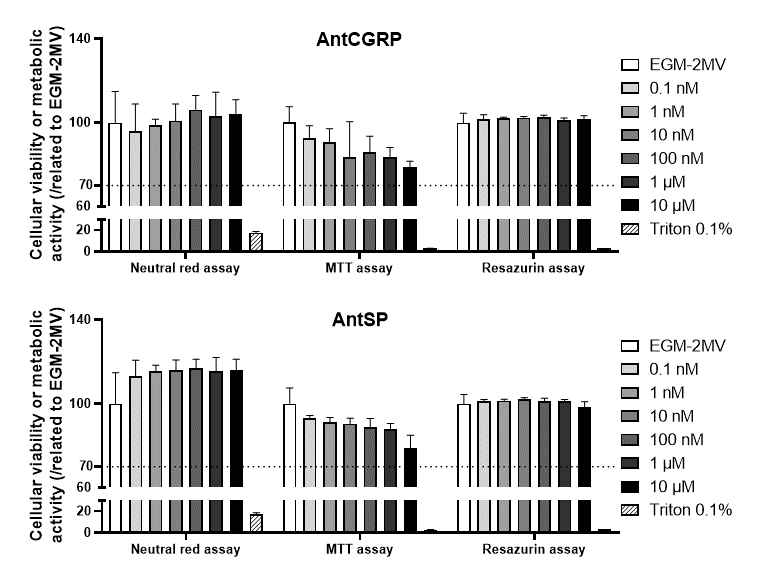


**Supplementary Figure 1.** Cytotoxicity tests performed for both BIBN4096 (AntCGRP) and SR140333 (AntSP) separately, at different concentrations in endothelial cells. Treatment with values under 70 % of cellular viability or metabolic activity is considered as cytotoxic.

**Supplementary Figure 2.** Original photomicrographies of western blots containing (B) the effect of sensory neurons (SNs) on Cx43 expression in endothelial cells and (F) the role of CGRP and SP using specific antagonists (BIBN4096 as AntCGRP, and SR140333 as AntSP).


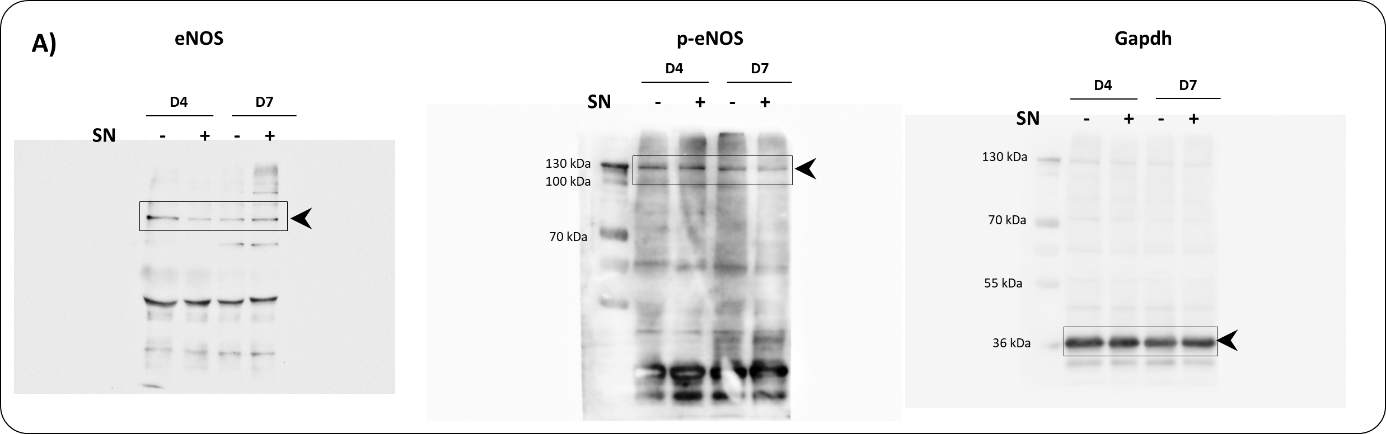


**Supplementary Figure 3.** Original photomicrographies of western blots containing (A) the effect of sensory neurons (SNs) on eNOS expression in endothelial cells.


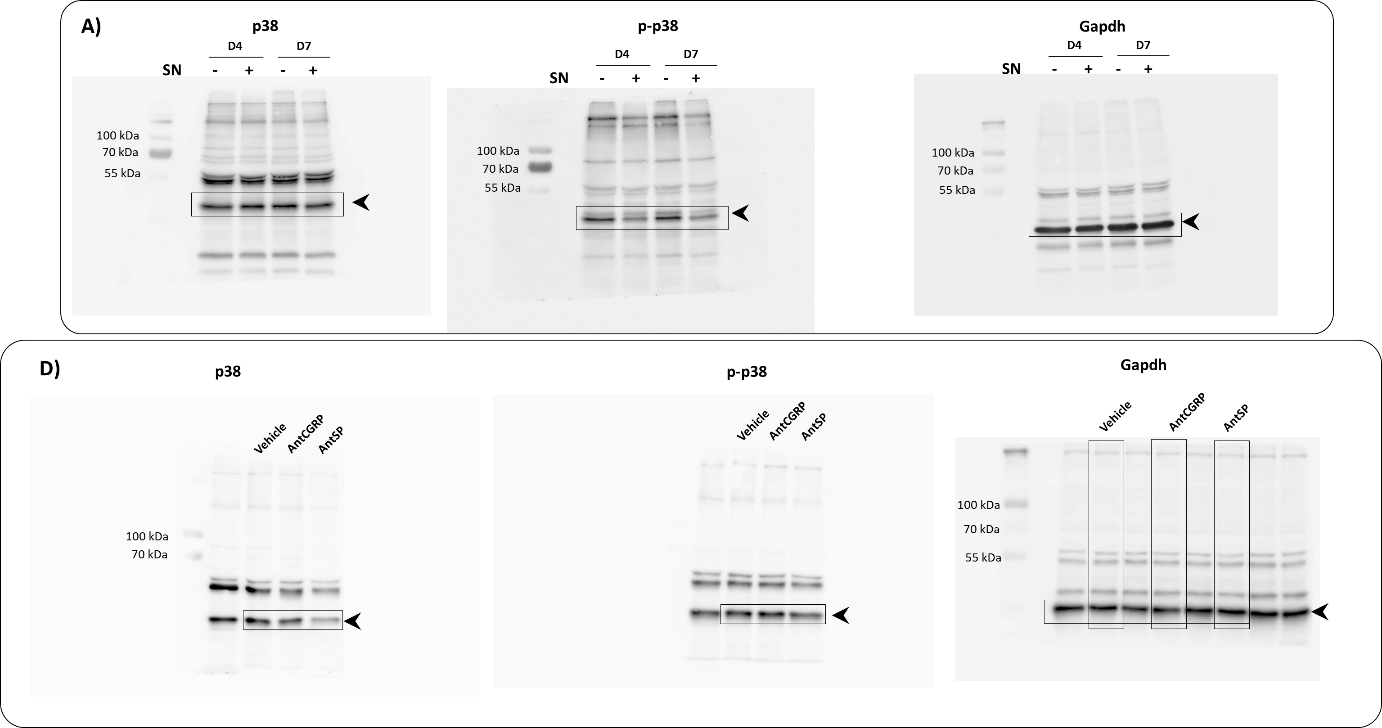


**Supplementary Figure 4.** Original photomicrographies of western blots containing (A) the effect of sensory neurons (SNs) on p38 expression in endothelial cells and (D) the role of CGRP and SP using specific antagonists (BIBN4096 as AntCGRP, and SR140333 as AntSP).

**Supplementary Figure 5.** Original photomicrographies of western blots containing (A) the effect of sensory neurons (SNs) on Erk1/2 expression and phosphorylation in endothelial cells, (D) the role of CGRP and SP using specific antagonists (BIBN4096 as AntCGRP, and SR140333 as AntSP), and (G) the effect of the neuropeptides CGRP and SP on endothelial cells.
